# Medium-chain triglycerides (8:0 and 10:0) increase muscle mass and function in frail older adults: a combined data analysis of clinical trials

**DOI:** 10.3389/fnut.2023.1284497

**Published:** 2023-12-04

**Authors:** Osamu Ezaki, Sakiko Abe

**Affiliations:** ^1^Institute of Women’s Health Science, Showa Women’s University, Tokyo, Japan; ^2^Department of Food and Nutrition, Faculty of Contemporary Human Life Science, Tezukayama University, Nara, Japan

**Keywords:** ketone body, ghrelin, frailty, sarcopenia, adipocyte triglyceride lipase, medium chain fatty acid, muscle mass, medium chain triglyceride

## Abstract

**Background:**

Three clinical trials have examined the chronic effects of medium-chain triglycerides (MCTs) on muscle mass and function in frail older adults (mean age 85 years old). However, significant increases in muscle mass and some muscle function relative to long-chain triglycerides (LCTs) have yet to be shown, possibly due to the small number of participants in each trial.

**Objective:**

We re-analyzed these previous clinical trials to clarify whether MCT supplementation can increase muscle mass and function.

**Analysis:**

After adding *post hoc* tests to the original report, we compared changes in measurement between the MCT and LCT groups in the first 2 trials and conducted a combined data analysis.

**Methods:**

In a combined data analysis, changes from baseline in measurements at the 3 months intervention in the MCTs- and LCTs-containing groups were assessed by analysis of covariance adjusted for baseline values of each measurement, age, sex, BMI, allocation to trial, habitual intakes in energy, protein, leucine, octanoic acid, decanoic acid, and vitamin D during the baseline period. The Mann–Whitney U test was used to analyze data on right and left knee extension times.

**Results:**

MCT supplementation for 3 months increased muscle function relative to LCT supplementation with and without an L-leucine (1.2 g) and vitamin D (cholecalciferol, 20 μg)-enriched supplement. In a combined data analysis (*n* = 29 in MCTs, *n* = 27 in LCTs), relative to supplementation with 6 g LCTs/day, supplementation with 6 g MCTs/day at dinner for 3 months significantly increased body weight (adjusted mean change from baseline: MCTs 1.2 vs. LCTs 0.2 kg, *p* = 0.023), right arm muscle area (MCTs 1.4 vs. LCTs-0.7 cm^2^, *p* = 0.002), left calf circumference (*p* = 0.015), right-hand grip strength (MCTs 1.6 vs. LCTs 0.3 kg, *p* = 0.017), right knee extension time (*p* = 0.021), left knee extension time (*p* = 0.034), walking speed (*p* = 0.002), and number of iterations in leg open and close test (*p* < 0.001) and decreased right triceps skinfold thickness (*p* = 0.016).

**Conclusion:**

In frail older adults, supplementation for 3 months with a low dose (6 g/day) of MCTs (C8:0 and C10:0) increased muscle mass and function. These findings indicate the potential for the practical use of MCTs in daily life in treating sarcopenia.

## 1 Introduction

Sarcopenia is characterized by the low skeletal muscle mass, strength, and function observed with aging (1). Exercise training and ingestion of adequate amounts of proteins are considered to be two primary lifestyles to slow the progression of sarcopenia (2). However, exercise training becomes difficult because of frailty, as does dietary intake, because of decreased appetite and digestive activity. Therefore, small amounts of foods or supplements that can be easily eaten and would maintain muscle mass and function are needed.

In an attempt to search for such nutrients, we found through a series of clinical trials that supplementation with a low dose of medium-chain triglycerides (MCTs) (6 g/day) may increase muscle mass and function without altering body weight in frail older adults (3–5). To compare the effects of the MCTs with those of long-chain triglycerides (LCTs), the changes from baseline between groups after interventions were compared in multiple groups (3, 4). In one trial, following 3 months of intervention, the increases in the numbers of iterations in the leg open and close test and the numbers of swallows were significantly greater in the MCT group than in the LCT group. However, the statistical significance of the increases in right arm muscle area (AMA), right-hand grip strength, right and left knee extension time, walking speed, and peak expiratory flow in the MCT group was not examined by a *post hoc* test because the fixed effect of the group by time was nonsignificant in a mixed-effect model, although these measurements in the MCT group showed a tendency to increase compared with those of LCT group (4). We re-analyzed the previous data by allowing the conduction of a *post hoc* test when the fixed effect of the group was significant, and the results were compared between trials to obtain a more accurate conclusion. In addition, combining the data in trials, a new analysis was conducted to compare the groups, including the MCTs and LCTs.

## 2 Materials and methods

### 2.1 Outline of clinical trials

Three clinical trials were conducted in a nursing home (Day Care SKY facility in Yokohama, Japan), and their protocols and methods were described in detail (3–7). We targeted all participants (mean age around 85 years) who resided in this nursing home and required special care from a helper. They were selected by the application of the exclusion criteria before enrollment as described previously (3–5). All trials were randomized, controlled (negative or positive control), single-blinded (or double-blinded for some measurements), and parallel-group trials. Each trial had a different purpose, group, and intervention period (Table 1). Six grams of MCTs (75% C8:0, 25% C10:0) per day were mixed with foods at mealtime in all trials. This article focuses on muscle mass and function in response to MCT supplementation. Therefore, we did not describe the cognition of the subjects in detail here.

**Table 1:** Outline of the clinical trials.

Trial	Group	*n* ^a^	*n* ^b^	Times of measurement	Analysis of the change
1		38	36	Baseline, 3.0-mo intervention	ANCOVA, per protocol analysis
	LD + MCT	13	13		
	LD + LCT	13	12		
	No supplements	12	11		
2		64	49	Baseline, 1.5-mo intervention, 3.0-mo intervention, washout (follow-up)	Mixed-effects model, intention-to-treat analysis
	LD + MCT	21	18		
	MCT	21	16		
	LCT	22	15		
3		40	0	Baseline, 1.5-mo intervention	ANCOVA, per protocol analysis
	MCT at breakfast	20	0		
	MCT at dinner	20	0		

a The number of participants at the enrollment and assignment to groups.

b The number of participants at the 3.0-mo intervention.

ANCOVA, analysis of covariance; LCT, long-chain triglyceride; LD, L-leucine + vitamin D; MCT, medium-chain triglyceride; mo, month.

The first trial (Trial 1), which started in September 2014 and ended in December 2014 (3, 6), was conducted to find a combination of nutrients that could treat sarcopenia. The participants (*n* = 38) were randomly allocated to three groups: the first group received an L-leucine (1.2 g) and vitamin D (cholecalciferol, 20 μg)-enriched supplement with 6 g of MCTs (LD + MCT); the second group received the same energy-matched supplement with 6 g of LCTs (LD + LCT); and the third group did not receive any supplements (no-supplements). The supplement and oils were taken at dinner. After three months of intervention, the LD + MCT participants had increased right-hand grip strength, walking speed, 10 s leg open and close test performance, and peak expiratory flow from baseline. In contrast, no significant improvements were observed in muscle function of the LD + LCT and no-supplements groups. Therefore, it was concluded that MCTs (6 g/d) played a pivotal role in the increase in muscle function in frail older individuals.

Trial 2 started in September 2016 and ended in February 2017 (4, 7). This trial aimed to clarify whether the favorable effects observed in the LD + MCT group in Trial 1 were due to MCT itself or the interaction between MCT and LD. The participants (*n* = 64) were randomly allocated to three groups: LD + MCT (positive control), MCT only (target), and LCT only (negative control). Each participant’s body weight, appendicular muscle mass, and function were assessed at four equally spaced time points: baseline, 1.5 and 3.0 months after initiation of the intervention (intervention), and 1.5 months after termination of the intervention (washout). MCT (6 g/d) supplementation alone increased the muscle function of frail older individuals, suggesting that MCT oil is a responsible nutrient involved in the favorable effects of LD + MCT on muscle function observed in Trial 1.

Trial 3 started in April 2019 and ended in June 2019 (5). In Trials 1 and 2, MCTs were given at dinnertime. This study aimed to determine the suitable timing of MCT supplementation during the day. The participants (*n* = 40) were randomly allocated to two groups, and we compared the effects of MCTs at breakfast or dinnertime for 1.5 months. Irrespective of ingestion at breakfast or dinnertime, supplementation with 6 g MCTs/day for 1.5 months increased muscle mass and function from baseline. However, a negative control group (i.e., the LCT group) was not set because the number of participants was insufficient.

### 2.2 Ethical approval

These clinical trials were approved by the Human Ethics Committee of Showa Women’s University (Nos. 14–10, 16–17, and 16–49) and by the Human Ethics Committee of Japan Society of Nutrition and Food Science (Approval No. 87). The procedures were conducted according to either the ethical standards of the institutional committee on human study or the Helsinki Declaration of 2000. Written informed consent was obtained from the participants and/or their family members in all trials.

### 2.3 Study products

The MCTs (75% C8:0 and 25% C10:0 from total fatty acids in oils) and LCTs (64% C18:1, 19% C18:2, and 9% C18:3 from total fatty acids in oils) were provided (Trial 1) by or purchased (Trials 2, 3) from Nisshin OilliO Group, Ltd. (Kanagawa, Japan). Six grams of MCTs (50 kcal; 8.3 kcal/g) or LCTs (54 kcal; 9 kcal/g) per day were mixed with foods such as steamed rice or miso soup at dinnertime (3, 4). The L-leucine (1.2 g) and vitamin D (cholecalciferol, 20 μg) (LD) -enriched supplement (Amino L40) was purchased from Ajinomoto Inc. (Tokyo, Japan). One tube (100 g, 30 kcal) of Amino L40 was given at the beginning of dinner (3).

### 2.4 Nutrient intake

The nursing care home served breakfast, lunch, and dinner daily. The individual participants’ habitual daily energy and nutrient intake during the baseline and intervention periods was calculated from data on food intakes for 7 separate days during each period using the Japanese Standard Tables of Food Composition as described previously (3–5).

### 2.5 Daily physical activity, including rehabilitation

Daily physical activities in this nursing home were as described previously (4). The individual daily activities and rehabilitation/exercise were not changed during the baseline and intervention periods.

### 2.6 Anthropometric measurements, muscle strength, and function measures

The items analyzed in each trial are described in detail in the original reports (3–5), and the measurements used in this article are briefly described below.

Measurement of the right mid-upper AMA was the best approach to assess the change in muscle mass in response to MCT supplementation among the anthropometric measures we have conducted. The AMA was calculated as follows: AMA = [mid-upper-arm circumference (AC) (cm) − π · triceps skinfold thickness (TSF) (cm)]^2^ / (4 · π) (8).

Maximal calf circumference (CC) was measured with each participant supine, with the left knee raised and the calf at right angles to the thigh. CC was used to assess calf muscle mass (9, 10).

The methods used to evaluate muscle function are restricted by the limited functional capabilities of frail older adults and the effect size of MCT supplementation. The methods that were feasible for frail older adults are described as follows.

For knee extension time, which is measured to examine muscle endurance of the quadriceps, the duration of holding each lower leg in the horizontal position was measured with the participant seated in a straight-backed chair.

For walking speed measurement, participants who could walk unaided were asked to walk for 10 m as fast as they could (3). Participants who had difficulty walking alone were asked to walk for 2.85 m with the support of parallel bars as fast as possible. Participants who could not walk for 2.85 m with the support of parallel bars used a walking aid to measure walking speed. Walking speed was calculated from the time and distance completed by each participant. The method to measure walking speed for individuals remained the same during the baseline and intervention periods.

For the leg open and close test, the number of iterations of opening and closing of the legs over a 10 s period with the participant sitting in a chair was calculated as described previously (11). Among the measurements we conducted, this test was the most sensitive for examining the impact of MCT supplementation on muscle function in frail older adults.

In people without lung disorders, peak expiratory flow was determined as an indicator of the strength of the respiratory muscles (12).

### 2.7 Statistical analysis

#### 2.7.1 Differences in statistical analyses between this article and the original reports

To compare results from Trial 1 with Trial 2, a similar statistical analysis of multiple comparisons between the 2 trials is presented in this article. Thus, in both trials, the adjusted mean changes for each baseline value and their relevant statistical analysis are shown in the figures (analysis of covariance [ANCOVA] in Trial 1, mixed-effects model in Trial 2).

In Trial 1, the numerical values of the non-adjusted (actual) mean changes were shown in tables of the original report (3), whereas, in Trial 2, those of the adjusted mean changes for each baseline value were shown in supplemental tables 1, 2 of the original report (4).

In the original report for Trial 2, when the fixed effect of the group was significant but that of the group by time was nonsignificant, *post hoc* tests were not performed for between-group and within-group analyses (4). However, in this article, *post hoc* tests were conducted because when the fixed effect of the group was significant, but that of the group by time interaction was nonsignificant, this indicates that there are statistically significant differences in the overall changes (mean of three time points) between the groups but not in their patterns. However, the third time point differed from the first and second times in that the third was in a follow-up period (washout) and not an intervention period. Thus, the overall changes (mean of three time points) between the groups were not compared because their comparisons were not meaningful. Therefore, in this article, when the fixed effect of the group was significant, *post hoc* tests with Bonferroni correction were performed for the between-group and within-group analyses. Thus, some of the results of the *post hoc* test presented in the figures of this article were not shown in supplemental tables 1, 2 of the original report (4).

#### 2.7.2 Combined analysis of clinical trials (Trials 1 and 2)

To interpret the impact of MCTs in Trials 1 and 2 together and increase the statistical power, the MCTs-containing group (*n* = 29, 84.6 + 6.0 years old) was created to combine the LD + MCT group (*n* = 13) in Trial 1 with the MCT group (*n* = 16) in Trial 2; also, the LCTs-containing group (*n* = 27, 86.1 + 5.4 years old) combined the LD + LCT group (*n* = 12) in Trial 1 with the LCT group (*n* = 15) in Trial 2. The number of participants in the group from each trial was that of participants who completed measurements at the 3.0-mo intervention, as described in Table 1. No duplications of participants from each trial were found in the combined data. Outcomes in a combination analysis were common to Trial 1 and Trial 2.

To eliminate the effects of possible confounding factors due to the different trials and genetic and environmental factors, changes in measurements from the baseline values at the 3 months intervention between the 2 groups were compared using one-way ANCOVA considering the following covariates: in model 1, the baseline value of the respective change; in model 2, additional adjustment for age, sex, and body mass index (BMI) at baseline and allocation to trials (Trial 1 or Trial 2); and in model 3, further additional adjustment for habitual intakes in energy (kcal/day), protein (g/day), leucine (g/day), octanoic acid (C8:0, mg/day), decanoic acid (C10:0, mg/day), and vitamin D (μg/day) during the baseline period. Additional covariates in model 3 were energy and nutrients that may relate to sarcopenia. Note that an assumption was made for adjusting the covariates for all outcomes; the group by the covariate interaction was assumed to be nonsignificant in these models: thus, linear regression curves between the covariate and the outcome in the 2 groups were assumed to be parallel in these models.

The reason for conducting ANCOVA to compare the 2 groups instead of an unpaired *t*-test was to adjust for possible confounding factors (13). However, when Levene’s test showed the variances of the changes in the 2 groups to be inhomogeneous, the Mann–Whitney U test was used to compare the changes between groups instead of ANCOVA. The Mann–Whitney U test was used to analyze the data on right and left knee extension times, but in this case, no adjustment was made for possible confounding factors. When the difference in changes between the 2 groups was statistically significant, the baseline and 3 months intervention values in each group were compared using the Wilcoxon signed-rank test.

#### 2.7.3 Additional details

Statistically significant differences in values between the groups with Bonferroni correction are shown in the figures as superscript letters, e.g., ^a, b^. The adjusted mean changes without a common superscript letter indicate a statistically significant difference between the groups at the same time point (*p* < 0.05). For example, X^a^, Y^b^, and Z^a^ indicate that X and Y, and Y and Z are statistically significant because there is no common superscript letter between X^a^ vs. Y^b^ and Y^b^ vs. Z^a^. In contrast, X and Z are not statistically significant because there is a common superscript letter ^a^ between X^a^ vs. Z^a^.

Statistical analyses were performed using the SPSS 20.0 and SPSS 28.0.1.0 (142) software programs (IBM, Chicago, IL). An α level of 0.05 was used to determine statistical significance.

## 3 Results

### 3.1 Comparison of MCT supplementation with (Trial 1) and without (Trial 2) the LD supplement

To examine the impact of MCTs relative to LCTs, the LD + MCT group was compared with the LD + LCT group (Trial 1), and the MCT group was compared with the LCT group (Trial 2). The data from Trial 3 were neither compared nor combined with others because there was no negative control group (LCT) and the intervention period was 1.5 months (the others were 3 months) (Table 1). Representative results are presented here.

#### 3.1.1 MCT supplementation did not alter right AC, decreased right TSF, and increased right AMA compared with LCT supplementation

**Figure 1:**
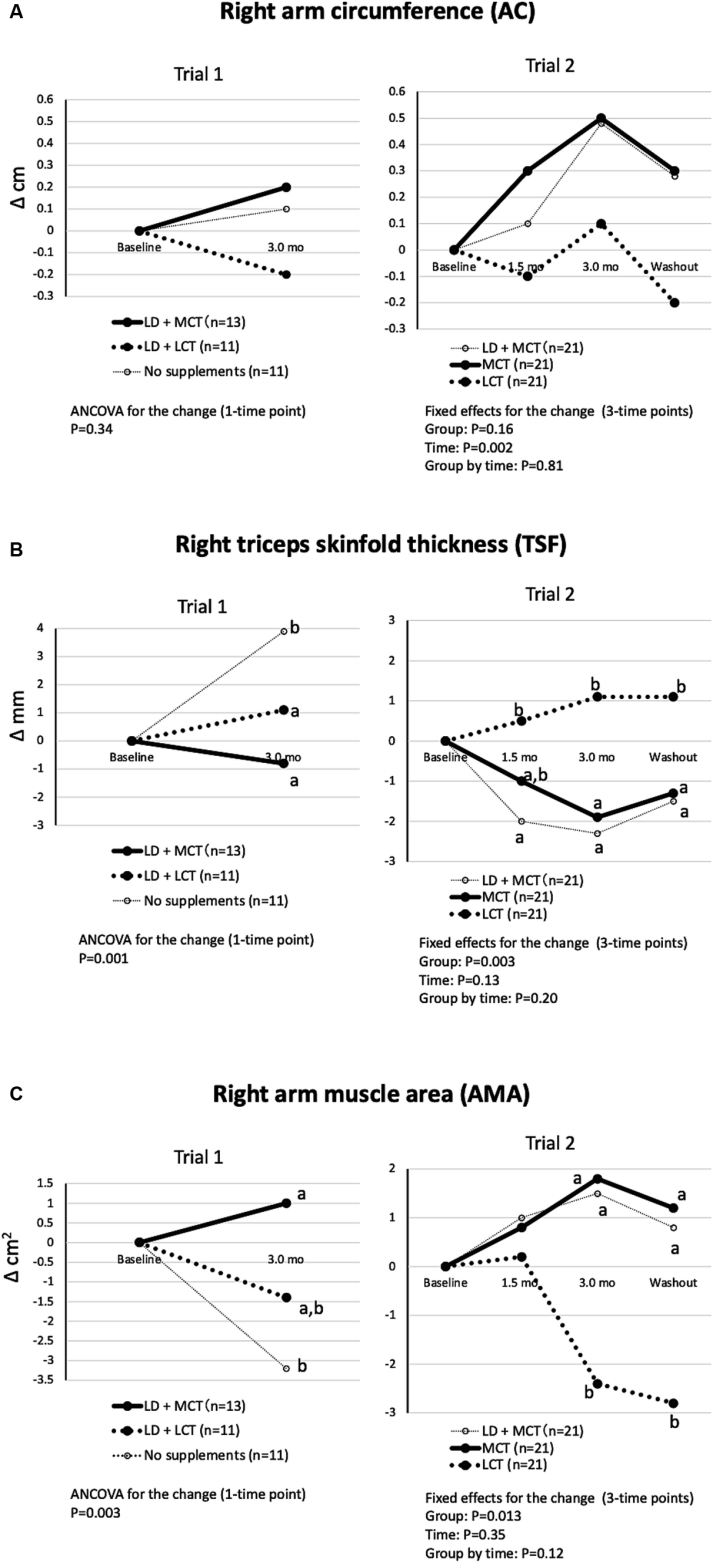
MCT supplementation did not alter right AC **(A)**, decreased right TSF **(B)**, and increased right AMA **(C)** compared with LCT supplementation. **(A)** Changes in right AC from baseline in Trials 1 and 2. Overall mean baseline right ACs were 21.9 cm (Trial 1) and 21.9 cm (Trial 2). **(B)** Changes in right TSF from baseline in Trials 1 and 2. Overall mean baseline right TSFs were 9.5 mm (Trial 1) and 10.6 mm (Trial 2). **(C)** Changes in right AMA from baseline in Trials 1 and 2. Overall mean baseline right AMAs were 28.7 cm^2^ (Trial 1) and 29.1 cm^2^ kg (Trial 2). The adjusted mean changes at a time point without a common letter indicate statistically significant differences between the groups, *p* < 0.05.

The mean adjusted changes in the right AC, TSF, and calculated AMA are shown in Figure 1. The differences in changes in the groups in right AC were nonsignificant in Trial 1 (ANCOVA, *p* = 0.34) and in Trial 2 (fixed effect of the group, *p* = 0.16) (Figure 1A). The differences in changes in the groups in right TSF were significant in Trial 1 (ANCOVA, *p* = 0.001) and in Trial 2 (fixed effect of the group, *p* = 0.003) (Figure 1B). The decrease in right TSF was greater in the MCT group than in the LCT group (Trial 2). The differences in changes in the groups in calculated right AMA were significant in Trial 1 (ANCOVA, *p* = 0.003) and in Trial 2 (fixed effect of the group, *p* = 0.013) (Figure 1C). The increase in the calculated right AMA at the 3.0-month intervention was greater in the MCT group than in the LCT group (Trial 2).

The differences in changes in the groups in calculated left AMA and right and left CCs were nonsignificant by ANCOVA and fixed effect of the group in Trials 1 and 2, respectively (data not shown in Figures) but did show a similar tendency to those in calculated right AMA (3, 4).

#### 3.1.2 MCT supplementation increased muscle function compared with LCT supplementation

**Figure 2:**
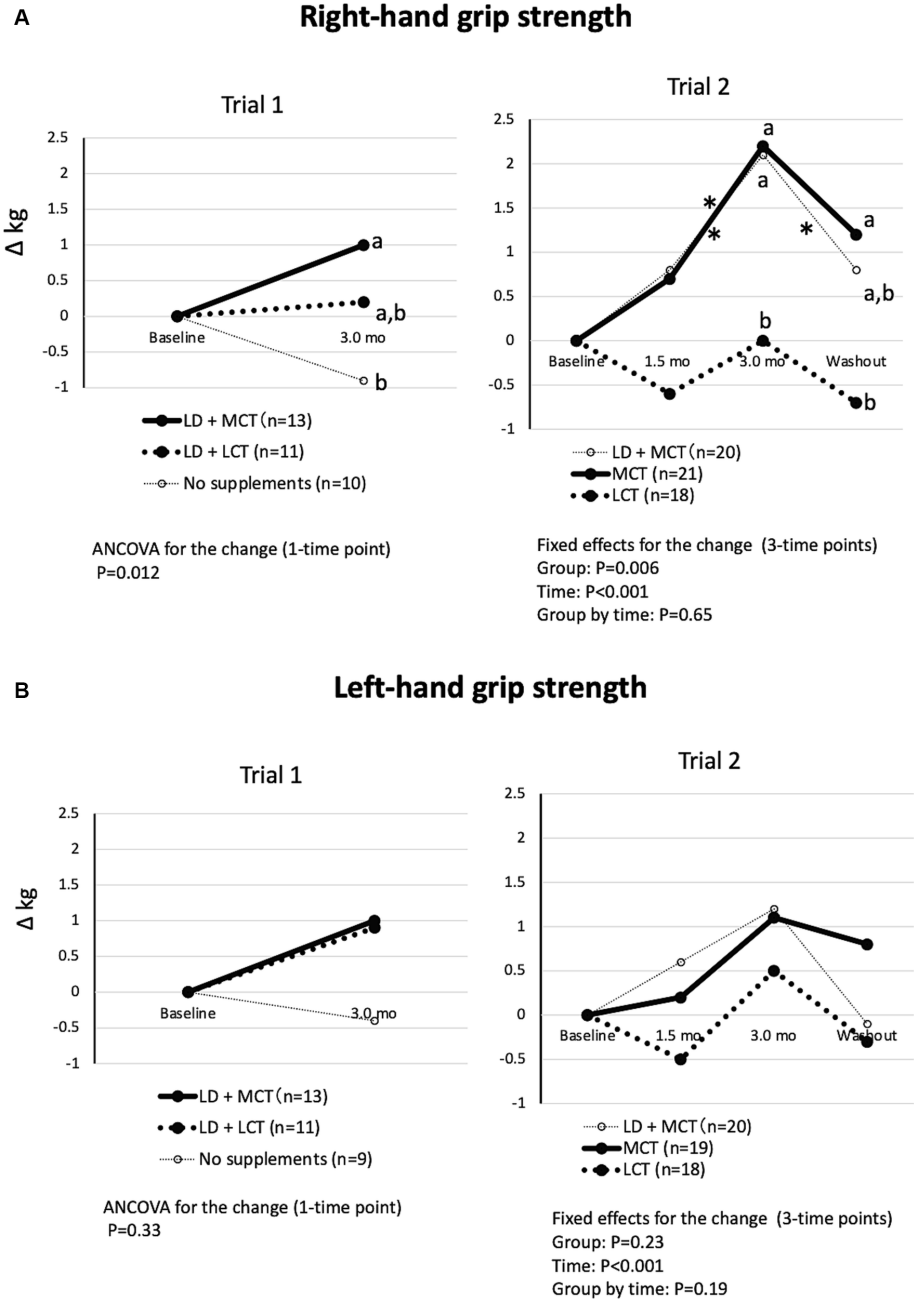
MCT supplementation increased right-hand grip strength **(A)** but not left-hand grip strength **(B)** compared with LCT supplementation. **(A)** Changes in right-hand grip strength from baseline in Trials 1 and 2. Overall mean baseline right-hand grip strengths were 10.7 kg (Trial 1) and 10.7 kg (Trial 2). **(B)** Changes in left-hand grip strength from baseline in Trials 1 and 2. Overall mean baseline left-hand grip strengths were 10.0 kg (Trial 1) and 10.6 kg (Trial 2). The adjusted mean changes at a time point without a common letter indicate statistically significant differences between the groups, *p* < 0.05. Asterisks indicate a statistically significant difference vs. at 3.0-mo intervention within the group, *p < 0.05.

The differences in changes in the groups in right-hand grip strength were significant in Trial 1 (ANCOVA, *p* = 0.012) and Trial 2 (fixed effect of the group, *p* = 0.006) (Figure 2A). In Trial 1, the increase in right-hand grip strength at the 3.0-month intervention was nonsignificant between the LD + MCT group and the LD + LCT group. However, in Trial 2, the increase in right-hand grip strength at the 3.0-month intervention and washout period was greater in the MCT group than in the LCT group. However, the change in left-hand grip strength did not differ between the groups (Figure 2B). This may be due to most participants’ left hand being non-dominant.

**Figure 3:**
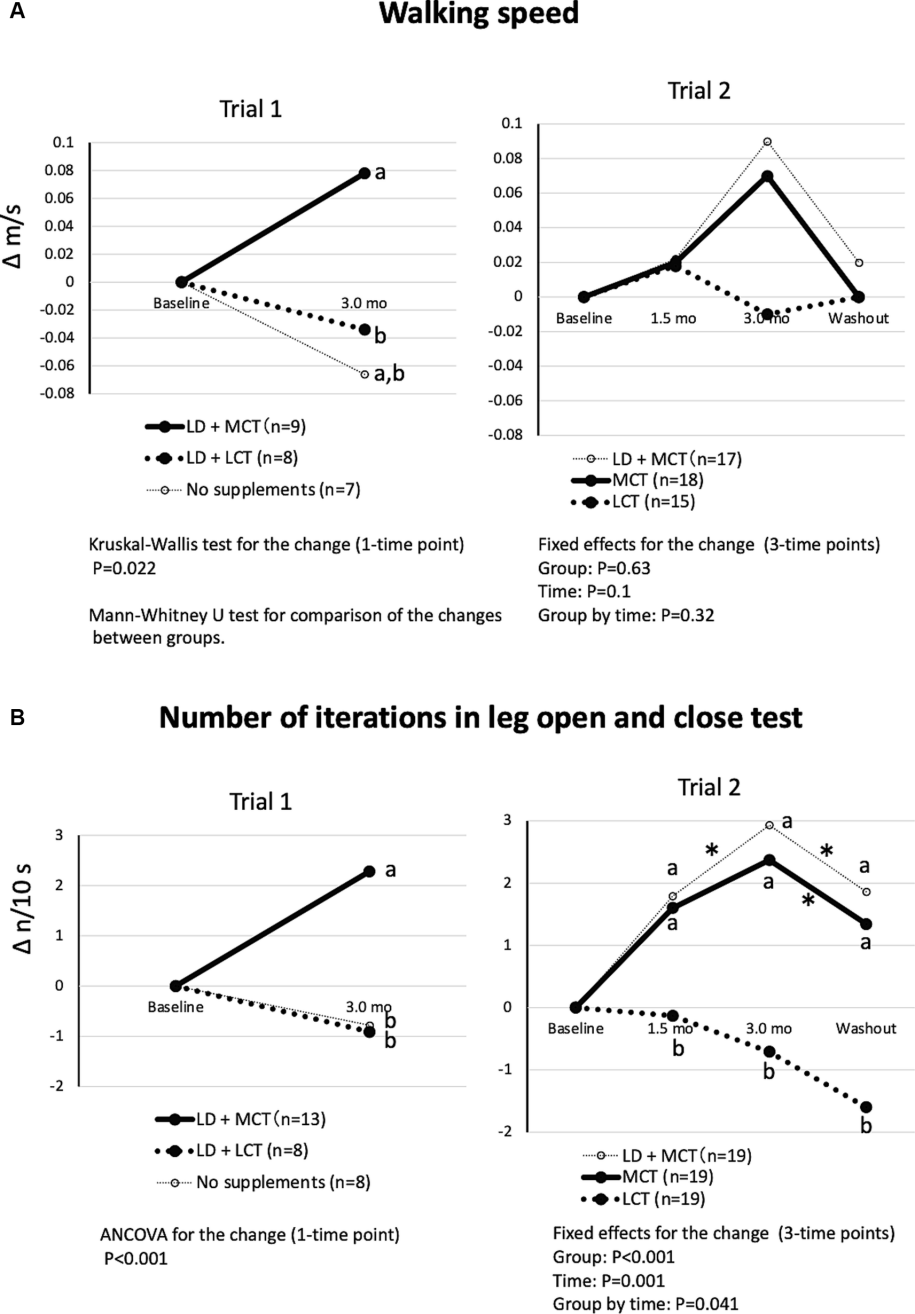
MCT supplementation increased walking speed **(A)** and number of iterations in the leg open and close test **(B)** compared with LCT supplementation. **(A)** Changes in walking speed from baseline in Trials 1 and 2. Overall mean baseline walking speeds were 0.50 m/s (Trial 1) and 0.52 m/s (Trial 2). **(B)** Changes in the number of iterations in the leg open and close test from baseline in Trials 1 and 2. Overall mean baseline numbers of iterations in the leg open and close test were 5.52 n/10 s (Trial 1) and 4.41 n/10 s (Trial 2). The adjusted mean changes at a time point without a common letter indicate statistically significant differences between the groups, *p* < 0.05. Asterisks indicate a statistically significant difference vs. at 3.0-mo intervention within the group, *p < 0.05.

The differences in changes in the groups in walking speed were significant in Trial 1 (Kruskal-Wallis test, *p* = 0.022) but not in Trial 2 (fixed effect of the group, *p* = 0.63) (Figure 3A). In Trial 1, the increase in walking speed at the 3.0-month intervention was greater in the LD + MCT group than in the LD + LCT group. Similarly, in Trial 2, the increase in walking speed at the 3.0-month intervention appeared to be greater in the MCT group than in the LCT group. However, a *post hoc* test was not conducted because the fixed effect of the group was nonsignificant.

The differences in changes in the groups in the number of iterations in the leg open and close test were significant in Trial 1 (ANCOVA, *p* < 0.001) and in Trial 2 (fixed effect of the group by time, *p* = 0.041) (Figure 3B). In Trial 1, the increase in the number of iterations at the 3.0-month intervention was greater in the LD + MCT group than in the LD + LCT group. Similarly, in Trial 2, the increases in the number of iterations at the 1.5- and 3.0-month interventions and the washout were greater in the MCT group than in the LCT group. At washout (1.5 months after termination of the intervention), the increase in the number of iterations returned to the level measured at the 1.5-month intervention.

As a result, similar effects of MCTs on muscle mass and function were observed in the presence (Trial 1) and absence (Trial 2) of the LD supplement. Then, to interpret the impact of MCTs in Trials 1 and 2 together and increase the statistical power, a combined data analysis was conducted.

### 3.2 Combined data analysis

Changes in measurements from baseline at the 3.0-month intervention between the MCTs- and LCTs-containing groups (combined groups from Trials 1 and 2) and their comparisons are shown in Table 2 (habitual energy and nutrient intakes), Table 3 (anthropometric measures) and Table 4 (muscle strength and functions).

**Table 2:** Combined data analysis in Trials 1 and 2; habitual energy and nutrient intakes at baseline and after the 3-mo intervention and their changes from baseline in the MCTs-containing (MCT or LD + MCT) and LCTs-containing (LCT or LD + LCT) groups (excluding supplements and added oils) (*n* = 56).^a^

Measure	Group	*n*	Baseline	3-mo intervention	Non-adjusted change	Adjusted change^c^ (model 1)	Adjusted change^d^ for trial
Energy, kcal/d	MCT, LD + MCT	29	1,379 ± 242	1,358 ± 230	−21 ± 113	−23 (−63, 17)	−22 (−63, 20)
	LCT, LD + LCT	27	1,406 ± 297	1,410 ± 294	4 ± 109	6 (−35, 47)	4 (−38, 47)
*p* value^b^					0.39	0.32	0.39
Energy, kJ/d	MCT, LD + MCT	29	5,873 ± 1,014	5,692 ± 966	−91 ± 476	−96 (−266, 72)	−91 (−263, 82)
	LCT, LD + LCT	27	5,894 ± 1,243	5,912 ± 1,232	18 ± 457	25 (−151, 200)	18 (−161, 197)
*p* value					0.39	0.32	0.38
Protein, en%	MCT, LD + MCT	29	16.4 ± 1.9	16.5 ± 2.2	0.1 ± 1.3	0.1 (−0.3, 0.5)	0.1 (−0.4, 0.5)
	LCT, LD + LCT	27	16.2 ± 2.2	15.8 ± 1.8	−0.4 ± 1.0	−0.4 (−0.9, −0.0)	−0.4 (−0.8, −0.0)
*p* value					0.14	0.08	0.13
Protein, g/d	MCT, LD + MCT	29	55.9 ± 7.4	55.2 ± 7.1	−0.7 ± 5.3	−0.7 (−2.4, 1.1)	−0.7 (−2.3, 1.0)
	LCT, LD + LCT	27	55.7 ± 9.4	55.0 ± 10.6	−0.6 ± 4.0	−0.7 (−2.5, 1.2)	−0.7 (−2.4, 1.0)
*p* value					0.97	0.98	0.98
Protein, g/(kg BW·d)	MCT, LD + MCT	29	1.35 ± 0.33	1.28 ± 0.25	−0.07 ± 0.15	−0.06 (−0.11, −0.02)	−0.07 (−0.12, −0.02)
	LCT, LD + LCT	27	1.30 ± 0.28	1.27 ± 0.29	−0.03 ± 0.10	−0.03 (−0.08, 0.01)	−0.03 (−0.08, 0.02)
*p* value					0.23	0.31	0.21
Leucine, g/d	MCT, LD + MCT	29	4.23 ± 0.59	4.12 ± 0.61	−0.11 ± 0.42	−0.11 (−0.26, 0.03)	−0.11 (−0.22, 0.01)
	LCT, LD + LCT	27	4.39 ± 0.66	4.20 ± 0.76	−0.19 ± 0.35	−0.18 (−0.33, −0.03)	−0.19 (−0.31, −0.07)
*p* value					0.43	0.53	0.32
EAAs, g/d	MCT, LD + MCT	29	21.7 ± 3.1	21.0 ± 3.1	−0.6 ± 2.1	−0.7 (−1.6, 0.3)	−0.6 (−1.5, 0.2)
	LCT, LD + LCT	27	22.0 ± 3.5	21.5 ± 4.3	−0.5 ± 2.9	−0.5 (−1.4, 0.5)	−0.5 (−1.4, 0.4)
*p* value					0.80	0.75	0.81
Carbohydrate, en%	MCT, LD + MCT	29	62.7 ± 5.7	61.1 ± 6.9	−1.6 ± 3.8	−1.6 (−2.8, −0.4)	−1.6 (−2.8, −0.4)
	LCT, LD + LCT	27	63.1 ± 6.1	63.5 ± 5.1	0.4 ± 2.5	0.4 (−0.8, 1.6)	0.4 (−0.9, 1.6)
*p* value					0.026	0.021	0.027
Carbohydrate, g/d	MCT, LD + MCT	29	218 ± 51	210 ± 52	−9 ± 23	−9 (−17, −8)	−9 (−17, −4)
	LCT, LD + LCT	27	224 ± 60	226 ± 57	1 ± 22	2 (−7, 10)	1 (−7, 10)
*p* value					0.10	0.08	0.10
Fat, en%	MCT, LD + MCT	29	20.9 ± 4.2	22.4 ± 4.8^**^	1.6 ± 2.9	1.6 (0.7, 2.5)	1.6 (0.7, 2.5)
	LCT, LD + LCT	27	20.8 ± 4.4	20.8 ± 4.0	0.0 ± 1.8	0.0 (−0.9, 1.0)	0.0 (−0.9, 1.0)
*p* value					0.024	0.021	0.023
Fat, g/d	MCT, LD + MCT	29	31.3 ± 5.5	33.1 ± 5.3	1.8 ± 4.2	1.7 (0.5, 3.0)	1.8 (0.4, 3.1)
	LCT, LD + LCT	27	31.8 ± 7.5	32.0 ± 7.1	0.2 ± 2.5	0.3 (−1.1, 1.6)	0.2 (−1.2, 1.6)
*p* value					0.11	0.11	0.11
Octanoic acid, mg/d	MCT, LD + MCT	29	74 ± 157	80 ± 158	6 ± 34	6 (−5, 18)	6 (−5, 18)
	LCT, LD + LCT	27	65 ± 119	70 ± 123	5 ± 26	5 (−6, 17)	5 (−6, 17)
*p* value					0.91	0.91	0.92
Decanoic acid, mg/d	MCT, LD + MCT	29	68 ± 71	78 ± 82	9 ± 36	9 (−3, 22)	9 (−4, 22)
	LCT, LD + LCT	27	83 ± 71	93 ± 82	10 ± 32	10 (−3, 23)	10 (−3, 23)
*p* value					0.89	0.96	0.89
Sodium, mg/d	MCT, LD + MCT	29	2,979 ± 725	3,074 ± 741	95 ± 327	103 (−2, 209)	95 (−11, 201)
	LCT, LD + LCT	27	2,657 ± 1,014	2,786 ± 1,003	129 ± 223	121 (11, 230)	130 (20, 240)
*p* value					0.65	0.82	0.65
Thiamin, mg/d	MCT, LD + MCT	29	1.1 ± 0.4	1.1 ± 0.4	0.0 ± 0.1	0.0 (−0.0, 0.1)	0.0 (−0.0, 0.1)
	LCT, LD + LCT	27	1.0 ± 0.4	1.0 ± 0.4	0.0 ± 0.1	0.0 (−0.0, 0.1)	0.0 (−0.0, 0.1)
*p* value					0.48	0.53	0.48
Pyridoxine, mg/d	MCT, LD + MCT	29	1.5 ± 0.6	1.5 ± 0.6	−0.0 ± 0.3	−0.0 (−0.1, 0.1)	−0.0 (−0.1, 0.1)
	LCT, LD + LCT	27	1.3 ± 0.6	1.3 ± 0.6	−0.0 ± 0.1	−0.0 (−0.1, 0.1)	−0.0 (−0.1, 0.1)
*p* value					0.97	0.91	0.97
Cyanocobalamin, μg/d	MCT, LD + MCT	29	3.8 ± 1.0	3.9 ± 1.2	0.1 ± 0.7	0.1 (−0.1, 0.3)	0.1 (−0.1, 0.3)
	LCT, LD + LCT	27	3.6 ± 1.1	3.7 ± 1.1	0.1 ± 0.3	0.1 (−0.1, 0.3)	0.1 (−0.1, 0.3)
*p* value					0.85	0.83	0.85
Vitamin D, μg/d	MCT, LD + MCT	29	5.0 ± 2.0	4.4 ± 1.2	-0.6 ± 1.0	−0.5 (−0.8, −0.3)	−0.6 (−0.8, −0.4)
	LCT, LD + LCT	27	4.6 ± 2.2	4.1 ± 1.6	-0.5 ± 0.9	−0.6 (−0.9, −0.4)	−0.6 (−0.8, −0.3)
*p* value					0.82	0.56	0.74

a Values are means ± SD or adjusted mean (95% CI). Difference from baseline by Wilcoxon signed-rank test, ^**^p < 0.01.

b *p* value represents the differences in the changes of variables between the 2 groups assessed by 1-factor ANCOVA.

c Adjusted for each baseline value (model 1).

d Adjusted for allocation to trial (Trial 1 or Trial 2).

En%, energy%; EAAs, essential amino acids; LCT, 6 g/d of long-chain triglycerides; LD + LCT, leucine and cholecalciferol-enriched supplement with 6 g/d of long-chain triglycerides; LD + MCT, leucine and cholecalciferol-enriched supplement with 6 g/d of medium-chain triglycerides; MCT, 6 g/d of medium-chain triglycerides.

**Table 3:** Combined data analysis in Trials 1 and 2; anthropometric measures at baseline and after the 3-mo intervention and their changes from baseline in the MCTs-containing (MCT or LD + MCT) and LCTs-containing (LCT or LD + LCT) groups (*n* = 56).^a^

Measure	Group	*n*	Baseline	3-mo intervention	Non-adjusted change	Adjusted change^c^ (model 1)	Adjusted change^d^ (model 2)	Adjusted change^e^ (model 3)
Body weight, kg	MCT, LD + MCT	29	43.4 ± 10.6	44.6 ± 10.1^***^	1.2 ± 1.7	1.2 (0.6, 1.8)	1.2 (0.6, 1.8)	1.2 (0.7, 1.8)
	LCT, LD + LCT	27	43.6 ± 5.8	43.8 ± 5.4	0.2 ± 1.6	0.2 (−0.4, 0.8)	0.3 (−0.4, 0.9)	0.2 (−0.4, 0.8)
*p* value^b^					0.026	0.021	0.036	0.023
BMI, kg/m^2^	MCT, LD + MCT	29	18.5 ± 3.4	19.1 ± 3.4^***^	0.6 ± 0.8	0.5 (0.3, 0.8)	0.6 (0.3, 0.8)	0.6 (0.3, 0.8)
	LCT, LD + LCT	27	19.2 ± 1.9	19.3 ± 1.7	0.1 ± 0.7	0.1 (−0.2, 0.4)	0.1 (−0.2, 0.4)	0.1 (−0.2, 0.4)
*p* value					0.027	0.042	0.031	0.039
Right AC, cm	MCT, LD + MCT	29	22.0 ± 3.6	22.4 ± 3.7^**^	0.5 ± 0.9	0.5 (0.1, 0.8)	0.4 (0.1, 0.8)	0.5 (0.1, 0.8)
	LCT, LD + LCT	26	22.8 ± 2.1	22.8 ± 1.8	−0.0 ± 0.9	−0.0 (−0.4, 0.3)	0.0 (−0.4, 0.4)	−0.0 (−0.3, 0.3)
*p* value					0.039	0.06	0.10	0.06
Left AC, cm	MCT, LD + MCT	29	21.9 ± 3.5	22.3 ± 3.5	0.4 ± 0.6	0.4 (0.2, 0.7)	0.4 (0.2, 0.7)	0.4 (0.1, 0.7)
	LCT, LD + LCT	26	22.4 ± 2.5	22.7 ± 2.2	0.3 ± 0.9	0.3 (−0.0, 0.6)	0.3 (−0.0, 0.5)	0.3 (0.0, 0.6)
*p* value					0.35	0.44	0.35	0.57
Right TSF, mm	MCT, LD + MCT	29	10.2 ± 5.2	8.8 ± 4.7^*^	−1.4 ± 3.1	−1.4 (−2.4, −0.4)	−1.2 (−2.1, −0.3)	−1.2 (−2.3, −0.2)
	LCT, LD + LCT	26	10.4 ± 4.9	11.3 ± 4.7	0.9 ± 2.9	0.9 (−0.1, 2.0)	0.7 (−0.3, 1.7)	0.8 (−0.3, 1.9)
*p* value					0.007	0.003	0.007	0.016
Left TSF, mm	MCT, LD + MCT	29	8.3 ± 5.3	7.6 ± 4.7	−0.7 ± 2.7	−0.7 (−1.7, 0.4)	−0.6 (−1.7, −0.4)	−0.4 (−1.6, 0.7)
	LCT, LD + LCT	26	8.4 ± 4.3	8.8 ± 4.8	0.4 ± 3.2	0.4 (−0.7, 1.5)	0.4 (−0.7, 1.5)	0.2 (−1.1, 1.4)
*p* value					0.18	0.16	0.20	0.51
Calculated right	MCT, LD + MCT	29	28.6 ± 6.0	30.3 ± 5.7^**^	1.7 ± 2.5	1.4 (0.5, 2.3)	1.3 (0.4, 2.1)	1.4 (0.6, 2.3)
AMA, cm^2^	LCT, LD + LCT	26	30.7 ± 5.3	29.8 ± 4.0	−0.9 ± 2.7	−0.7 (−1.6, 0.3)	−0.5 (−1.3, 0.4)	−0.7 (−1.6, 0.2)
*P* value					<0.001	0.002	0.006	0.002
Calculated left	MCT, LD + MCT	29	29.1 ± 5.1	30.8 ± 5.4	1.7 ± 3.4	1.6 (0.5, 2.7)	1.6 (0.5, 2.7)	1.5 (0.3, 2.6)
AMA, cm^2^	LCT, LD + LCT	26	30.8 ± 4.7	31.1 ± 5.1	0.4 ± 2.5	0.5 (−0.7, 1.6)	0.5 (−0.6, 1.7)	0.7 (−0.6, 1.9)
*p* value					0.10	0.16	0.19	0.38
Right CC, cm	MCT, LD + MCT	28	28.8 ± 4.3	29.0 ± 4.4	0.2 ± 1.0	0.2 (−0.2, 0.6)	0.3 (−0.2, 0.7)	0.4 (−0.1, 0.8)
	LCT, LD + LCT	25	28.9 ± 3.1	28.9 ± 2.8	−0.1 ± 1.2	−0.0 (−0.5, 0.4)	−0.1 (−0.5, 0.4)	−0.2 (−0.7, 0.3)
*p* value					0.34	0.36	0.32	0.13
Left CC, cm	MCT, LD + MCT	28	28.5 ± 4.5	28.8 ± 4.5	0.3 ± 0.8	0.3 (−0.2, 0.7)	0.4 (−0.1, 0.8)	0.5 (0.0, 0.9)
	LCT, LD + LCT	25	28.5 ± 2.8	28.3 ± 2.4	−0.2 ± 1.5	−0.2 (−0.6, 0.3)	−0.3 (−0.7, 0.2)	−0.4 (−0.9, 0.1)
*p* value					0.16	0.16	0.06	0.015

a Values are means ± SD or adjusted mean (95% CI). Difference from baseline by Wilcoxon signed-rank test, ^*^p < 0.05, ^**^p < 0.01, ^***^p < 0.001.

b *p* value represents the differences in the changes of variables between the 2 groups assessed by 1-factor ANCOVA.

c Adjusted for each baseline value (model 1).

d Additionally adjusted for age, sex, BMI, and allocation to trial (Trials 1 or 2) (model 2).

e Further additionally adjusted for intakes in energy (kcal/day), protein (g/day), leucine (g/day), octanoic acid (C8:0, mg/day), decanoic acid (C10:0, mg/day), and vitamin D (μg/day) during the baseline period (model 3).

AC, arm circumference; AMA, arm muscle area; BMI, body mass index; CC, calf circumference; LCT, 6 g/d of long-chain triglycerides; LD + LCT, leucine and cholecalciferol-enriched supplement with 6 g/d of long-chain triglycerides; LD + MCT, leucine and cholecalciferol-enriched supplement with 6 g/d of medium-chain triglycerides; MCT, 6 g/d of medium-chain triglycerides; TSF, triceps skinfold thickness.

**Table 4:** Combined data analysis in Trials 1 and 2; muscle strength and function at baseline and after the 3-mo intervention and their changes from baseline in the MCTs-containing (MCT or LD + MCT) and LCTs-containing (LCT or LD + LCT) groups (*n* = 56).^a^

Measure	Group	*n*	Baseline	3-mo intervention	Non-adjusted change	Adjusted change^c^ (model 1)	Adjusted change^d^ (model 2)	Adjusted change^e^ (model 3)
Right-hand grip	MCT, LD + MCT	29	11.3 ± 7.6	13.0 ± 7.2^***^	1.7 ± 2.0	1.7 (1.1, 2.3)	1.7 (1.1, 2.3)	1.6 (0.9, 2.2)
strength, kg	LCT, LD + LCT	25	11.7 ± 5.1	11.8 ± 4.9	0.2 ± 1.3	0.2 (−0.5, 0.8)	0.2 (−0.5, 0.8)	0.3 (−0.4, 1.0)
*p* value^b^					0.002	0.001	0.002	0.017
Left-hand grip	MCT, LD + MCT	27	10.2 ± 4.6	11.4 ± 4.5	1.3 ± 2.6	1.2 (0.3, 2.1)	1.1 (0.1, 2.0)	0.8 (−0.2, 1.8)
strength, kg	LCT, LD + LCT	25	10.8 ± 5.4	11.6 ± 5.5	0.8 ± 2.3	0.8 (−0.1, 1.8)	1.0 (0.0, 2.0)	1.3 (0.3, 2.3)
*p* value					0.51	0.57	0.96	0.54
Right knee	MCT, LD + MCT	28	74 ± 40	105 ± 29^***^	31 ± 35			
extension time, s	LCT, LD + LCT	24	70 ± 43	73 ± 42	3 ± 46		Not applicable	
*p* value^f^					0.021			
Left knee	MCT, LD + MCT	27	78 ± 42	106 ± 32^**^	28 ± 36			
extension time, s	LCT, LD + LCT	25	69 ± 42	71 ± 44	2 ± 46		Not applicable	
*p* value					0.034			
Walking speed,	MCT, LD + MCT	23	0.637 ± 0.409	0.706 ± 0.427^**^	0.069 ± 0.124	0.068 (0.022, 0.113)	0.076 (0.032, 0.119)	0.084 (0.040, 0.128)
m/s	LCT, LD + LCT	20	0.436 ± 0.257	0.420 ± 0.285	−0.016 ± 0.073	−0.014 (−0.062, 0.035)	−0.023 (−0.070, 0.024)	−0.033 (−0.080, 0.015)
*p* value					0.011	0.020	0.005	0.002
Legs open and	MCT, LD + MCT	28	4.14 ± 3.13	6.48 ± 3.90^***^	2.34 ± 1.88	2.32 (1.67, 2.97)	2.38 (1.71, 3.06)	2.40 (1.70, 3.11)
close test, n/10 s	LCT, LD + LCT	22	5.80 ± 2.89	4.96 ± 2.59^*^	−0.84 ± 1.35	−0.82 (−1.56, −0.08)	−0.89 (−1.66, −0.12)	−0.92 (−1.73, −0.11)
*p* value					< 0.001	<0.001	<0.001	<0.001
Peak expiratory	MCT, LD + MCT	25	189 ± 83	220 ± 82^**^	31 ± 48	32 (13, 52)	32 (13, 51)	34 (13, 55)
flow, L/min	LCT, LD + LCT	26	173 ± 66	179 ± 75	6 ± 50	5 (−14, 24)	5 (−13, 23)	3 (−18, 24)
*p* value					0.08	0.049	0.048	0.06

a Values are means ± SD or adjusted mean (95% CI). Difference from baseline by Wilcoxon signed-rank test, ^*^p < 0.05, ^**^p < 0.01, ^***^p < 0.001.

b *p* value represents the differences in the changes of variables between the 2 groups assessed by 1-factor ANCOVA.

c Adjusted for each baseline value (model 1),

d Additionally adjusted for age, sex, BMI, and allocation to trial (Trials 1 or 2) (model 2).

e Further additionally adjusted for intakes in energy (kcal/day), protein (g/day), leucine (g/day), octanoic acid (C8:0, mg/day), decanoic acid (C10:0, mg/day), and vitamin D (μg/day) during the baseline period (model 3).

f The changes in right and left knee extension times were assessed by a nonparametric Mann–Whitney U test.

LCT, 6 g/d of long-chain triglycerides; LD + LCT, leucine and cholecalciferol-enriched supplement with 6 g/d of long-chain triglycerides; LD + MCT, leucine and cholecalciferol-enriched supplement with 6 g/d of medium-chain triglycerides; MCT, 6 g/d of medium-chain triglycerides.

#### 3.2.1 Habitual energy and nutrient intakes in a combined data analysis

Note that to examine the effects of the supplements on habitual energy and nutrient intakes, the energy and nutrients in the supplements were not included in Table 2. There was a statistically significant difference between the 2 groups in the energy of fat and carbohydrate intake from total energy; MCT supplementation increased the fat energy % by 1.6%, whereas it decreased carbohydrates by-1.6% compared with LCT supplementation. These differences were also observed after adjusting each baseline value or allocation to trials (Table 2). The increase in fat energy was due to increased fat and decreased carbohydrate intakes in the MCTs-containing group. Similar changes, but nonsignificant changes, were observed after MCT supplementation in each of Trial 1 (3) and Trial 2 (data not shown). The physiological and clinical effects of this slight increase in fat energy % are unclear. It may reflect the increased fat oxidation observed after MCT supplementation (14). No differences in changes in the 2 groups were observed in the intakes of energy and other nutrients.

#### 3.2.2 Anthropometric measures, muscle strength, and functions in a combined data analysis

In Tables 3, 4, possible confounding factors were adjusted by 3 stepwise adjustments (models 1, 2, 3) for covariates by ANCOVA (see Materials and methods for details). In Table 3, the increase in right AC between the 2 groups was significant without the adjustment (*p* = 0.039) but nonsignificant after the adjustment of its baseline value (model 1) (*p* = 0.06), whereas that in left CC became significant after the adjustment of energy and nutrient intakes (model 3) (*p* = 0.015). In Table 4, the significance of peak expiratory flow varied; it was significant in models 1 and 2 but not in the non-adjusted model and model 3. The presence of statistical significance in other measurements remained the same after the adjustments.

In the fully adjusted model (model 3), statistically significant increases from baseline in the changes after the 3.0-month intervention in measurements in the MCTs-containing group, relative to the LCTs-containing group, were manifested in body weight (adjusted mean change from baseline: MCTs 1.2 kg vs. LCTs 0.2 kg, *p* = 0.023), BMI (MCTs 0.6 kg/m^2^ vs. LCTs 0.1 kg/m^2^, *p* = 0.039), right AMA (MCTs 1.4 cm^2^ vs. LCTs-0.7 cm^2^, *p* = 0.002), left CC (MCTs 0.5 cm vs. LCTs-0.4 cm, *p* = 0.015), right-hand grip strength (MCTs 1.6 kg vs. LCTs 0.3 kg, *p* = 0.017), walking speed (MCTs 0.084 m/s vs. LCTs-0.033 m/s, *p* = 0.002), and number of iterations in leg open and close test (MCTs 2.40 n/10 s vs. LCTs-0.92 n/10 s, *p* < 0.001). In contrast, statistically significant decreases were observed in the right TSF (MCTs-1.2 mm vs. LCTs 0.8 mm, *p* = 0.016). Increases in the MCTs-containing group were also observed in right knee extension time (MCTs 31 s vs. LCTs 3 s, *p* = 0.021) and left knee extension time (MCTs 28 s vs. LCTs 2 s, *p* = 0.034) by the Mann–Whitney U test.

## 4 Discussion

A combined data analysis showed that relative to supplementation with 6 g LCTs/day, supplementation with 6 g MCTs/day for 3 months statistically significantly increased body weight, BMI, muscle mass (right AMA, left CC), and function (right-hand grip strength, right and left knee extension times, walking speed, and number of iterations in leg open and close test) and decreased fat mass (right triceps skinfold thickness) (Tables 3, 4). This is the first report to show a statistically significant increase in muscle mass in response to MCT supplementation relative to LCT supplementation.

Three factors, (1) the number of participants, (2) the selection of confounding factors, and (3) the selection of outcomes, affect the contributions of independent variables to the outcomes. In a combined data analysis, we increased the number of participants (*n* = 56) and considered 11 possible confounding factors. The advantages of covariate-adjusted analysis were reviewed previously (13). Also, we conducted 7 outcome measurements of muscle function in Trials 1 and 2 and performed a combined data analysis for each measurement. Although the precision of the measures for body weight, muscle mass, and function to detect their small changes in frail old adults was unclear, incorporating these 3 appropriate factors in a combined analysis may have increased the statistical power to detect the difference in outcomes between the 2 groups.

A dose of 6 g/day is much less than that aimed to increase ketone bodies in the blood (usually 20 ~ 40 g/day of MCTs) (15). A 6 g/day intake of MCTs corresponds to about 4% of habitual energy intake (overall mean habitual energy intake was 1,430 kcal/day), 15% of habitual fat intake (overall mean fat intake was 39 g/day), and 0.14 g/kg body weight (overall mean body weight was 42.6 kg) in this population (overall mean age 85 years old). The significant increase in body weight in the MCTs-containing group is somewhat surprising because MCTs have been considered to reduce body weight and total body fat relative to LCTs (16, 17). The increase in muscle mass may contribute to maintaining or increasing body weight despite a decrease in fat mass in frail older adults.

### 4.1 Rationale for combining groups from different trials

Because MCT supplementation for 3 months increased muscle function similarly relative to LCT supplementation in Trials 1 and 2, irrespective of the LD supplement given (Figures 1–3), the MCTs-containing group (*n* = 29) was created to combine the LD + MCT group in Trial 1 with the MCT group in Trial 2; also, the LCTs-containing group (*n* = 27) combined the LD + LCT group in Trial 1 with the LCT group in Trial 2, and these 2 groups were compared. Thus, the MCTs- and LCTs-containing groups have a similar number of participants from Trials 1 and 2 (balanced data) and are comparable. However, participants from Trial 1 received an additional supplement of LD. To examine the possible effects of LD and the difference in the trials on the outcomes in the combined data analysis, allocation to trial (Trials 1 or 2) was adjusted for a covariate. There were almost no effects on either the changes in each group or on the difference in changes between the 2 groups for any of the measurements after additional adjustment for “allocation to trial” in the non-adjusted model in energy and nutrient intakes (Table 2) and in the non-adjusted model or model 1 in anthropometric measures and muscle function, respectively (Supplementary Tables S1, S2). However, there was an increase in the statistical power of the MCT effect with this adjustment in the measurement of peak expiratory flow: the *p* value of the difference in the non-adjusted changes between the 2 groups was 0.08, whereas that in the adjusted change for “allocation to trial” was 0.045; that in the adjusted change for baseline value was 0.049, whereas that in the adjusted change for baseline value and “allocation to trial” was 0.028 (the last line in Supplementary Table S2). These results suggest that the LD supplement or “allocation to trial” did not affect most outcomes but somehow affected those of the peak expiratory flow for unknown reasons. This may be one of the reasons that the significance of changes between the 2 groups in the peak expiratory flow varied in the models (Table 4).

### 4.2 Confounding factors

Overall, the confounding factors we examined showed minimal effects on the outcomes (Tables 3, 4). However, an increase in left CC in the MCTs-containing group relative to the LCTs-containing group became significant after the additional adjustment of habitual energy and several nutrient intakes during the baseline period (MCTs 0.5 cm [0.0, 0.9] vs. LCTs-0.4 cm [−0.9, 0.1], *p* = 0.015) (Table 3). A similar tendency was observed in right CC, but this was nonsignificant (MCTs 0.4 cm [−0.1, 0.8] vs. LCTs-0.2 cm [−0.7, 0.3], *p* = 0.13). The following 3 covariates in model 3 contributed significantly to the change in the left CC: the estimated partial regression coefficient B for BMI at baseline was 0.324 (SE 0.092, *p* = 0.001); energy intake during baseline was 0.003 (SE 0.001, *p* = 0.039); and left CC at baseline was-0.306 (SE 0.077, *p* < 0.001) (data not shown). Although these mixed confounding factors may affect the outcome, their clinical significance is unclear.

### 4.3 Is increased muscle mass required for increased muscle strength and function in response to MCT supplementation?

Although a decrease in muscle mass is essential to the definition of sarcopenia (12), physiologically, an increase in muscle mass might not necessarily result in increased muscle strength and function. As observed in exercise training, improved functions of the mitochondria in skeletal muscles (18), neuromuscular junctions (19), and the brain (20), in addition to increasing muscle mass (i.e., increases in the number or size of type I or type II fibers) (21, 22), may increase muscle strength and function.

Kojima et al. recently reported that in healthy middle-aged and older adults (mean age 68 years old), supplementation with 6 g/day MCTs (C8:0 or C10:0) combined with moderate-intensity aerobic exercise for 3 months significantly increased muscle function (right knee extension strength) but did not alter skeletal muscle mass (overall mean skeletal muscle mass was 23 kg) compared with 6 g/day LCTs combined with aerobic exercise (23). Also, Mutoh et al. reported that supplementation with 18 g MCTs/day in healthy older adults (mean age 70 years old) for 3 months resulted in the participants showing better balance ability (one of the muscle functions) relative to those receiving a placebo (24). These findings suggested that MCTs could increase muscle function without increasing muscle mass in healthy older adults and that an increase in muscle mass by MCT supplementation might manifest in subjects whose muscle mass at baseline is very low (overall mean muscle mass was 16 kg), as is observed in frail older adults. It is also conceivable that an increase in muscle function may eventually increase muscle mass in frail older adults. However, precision in the measurement of muscle mass and functions is essential and is required for these studies.

Cognition was also improved after MCT supplementation in all trials (5–7). Therefore, improved cognition might affect muscle function (20). However, in the LD + MCT group in Trial 1, there was no significant correlation between changes in cognition test scores and changes in muscle mass and function (6). In Trial 2, the increases in peak expiratory flow and the number of swallows in 30 s persisted for 1.5 months after termination of the intervention (washout) (4). In contrast, the increases in cognition score returned to the baseline levels (7). These data suggested that the increase in cognition did not directly mediate increases in muscle function. However, it is conceivable that the motor and cognition areas in the brain were activated by a similar mechanism after MCT supplementation.

### 4.4 Acyl-ghrelin (an active form) may mediate increased muscle mass in response to MCT supplementation

After MCT supplementation in frail older adults, slight but significant increases in body weight and muscle mass with a decrease in fat mass were observed without a concomitant increase in energy intake. These findings indicated that the energy in fat tissues might be used for muscle tissues, and an anabolic effect was seen on muscle mass. This anabolic effect is similar to that of growth hormone (GH) (25). Interestingly, there is a close relationship between MCTs and GH via ghrelin.

Activated ghrelin (acyl-ghrelin) formed by proghrelin and octanoic acid (C8:0) via ghrelin-O-acyltransferase in the stomach, enters the blood circulation and stimulates GH release in the brain (26) and GH increases muscle mass (the MCTs/Ghrelin/GH hypothesis). MCT supplementation increases blood acyl-ghrelin concentrations in humans (27–29). Supplementation with 6 g MCTs/day for 2 weeks increased acyl-ghrelin from 20 fmol/mL (baseline) to 40 fmol/mL (2-fold increase) and decreased desacyl-ghrelin (an inactive form) from 210 fmol/mL (baseline) to 160 fmol/mL in patients with anorexia nervosa (mean age, 26.4 years; BMI, 13.0 kg/m^2^) (28). A lower dose of MCTs was able to increase acyl-ghrelin: single oral ingestion of 3 g of MCT (100% C8:0) with enteral nutrition formula in cachectic patients increased plasma acyl-ghrelin, and the 2 weeks administration of MCTs increased appetite score, body weight, and serum albumin and insulin-like growth factor-1 (but not GH) concentrations compared with their baseline levels (27). Considering the effects of a low dose of MCTs on acyl-ghrelin concentration, acyl-ghrelin may mediate the increases in muscle mass and function in response to MCT supplementation in frail older adults. Because a decrease in GH secretion was observed with aging, MCT supplementation in older adults might be timely (30). However, it has not been shown that MCT supplementation increases GH secretion.

Many reports show that acute acyl-ghrelin injection (or infusion) increased GH concentration, appetite, and food intake and that chronic acyl-ghrelin injection increased body weight, as listed in a supplemental table of a review (31). Chronic effects of acyl-ghrelin for years are mimicked by growth hormone secretagogues receptor agonists, which require no injections. Indeed, the impact of these oral agonists on body composition has been reported in three clinical trials (32–34), all of which showed increases in body weight and fat-free mass with no change in fat mass. These data suggested that acyl-ghrelin might lead to an increase in muscle mass but not a decrease in fat mass. Because MCT supplementation decreases fat mass, other mechanisms may be involved in reducing fat mass in response to MCT supplementation.

### 4.5 Effects of MCT supplementation on heart and skeletal muscles in patients with triglyceride deposit cardiomyovasculopathy (TGCV) or neutral lipid storage disease with myopathy (NLSDM)

Because in both TGCV and NLSDM (primary TGCV and NLSDM might be the same disease because the responsive gene is identical), lipid accumulation in cardiomyocytes and myocytes may cause heart failure and muscle atrophy, respectively (35, 36), MCTs, which decrease fat accumulation, have been considered a promising candidate for treating patients suffering from these conditions (37).

TGCV is a rare intractable disease in which impaired intracellular lipolysis results in massive triglyceride accumulation in the myocardium and coronary arteries, caused by genetic (primary) or acquired dysfunction of adipose triglyceride lipase (ATGL) (36). This leads to heart failure and ischemic heart disease (38). Long-chain fatty acids (LCFAs) entering cardiomyocytes are re-esterized to LCTs to form an energy pool in cells and are immediately hydrolyzed by intracellular lipases such as ATGL. In TGCV, the impaired intracellular lipolysis of LCTs results in ectopic deposition and loss of LCFA supply to mitochondria, leading to lipotoxicity and energy failure in cardiomyocytes and coronary smooth muscle cells. Because cytoplasmic MCTs were degraded at an average rate, whereas LCTs remained undegraded in fibroblasts from NLSDM patients (39), MCTs might be effective in treating TGCV. Indeed, cardiac imaging tests showed that a tricaprin diet (100% C10:0 with MCTs corresponding to 80% of total fat intake) reduced triglyceride accumulation and improved metabolism of LCFAs and left ventricular function in ATGL knockout (KO) mice compared with the ATGL KO mice fed the control diet (40). Recently, a randomized controlled MCT trial for idiopathic TGCV in humans (*n* = 17) was reported (41). In agreement with the results in ATGL KO mice, compared with the placebo (*n* = 8), supplementation with a 1.5 g/day of CNT-01 (100% C10:0) for 8 weeks (*n* = 9) improved myocardial lipolysis (*p* = 0.035) as estimated by iodine-123-beta-methyl-p-iodophenylpentadecanoic acid scintigraphy. However, decreases in TGCV severity scores (lower score indicates better outcome) between the 2 groups were nonsignificant, probably due to the short term of the intervention period. The adjusted mean changes from baseline (95% CI) were as follows: TGCV severity symptom score [CNT-01-2.78 (−4.61, −0.94) vs. placebo-0.75 points (−3.38, 1.88)] and TGCV severity ADL score [CNT-01-2.67 (−6.47, 1.14) vs. placebo-0.88 points (−3.30, 1.55)] (41).

In contrast, in a 26-year-old female patient with NLSDM caused by a mutation of ATGL without cardiac involvement, no beneficial effects on progressive muscle weakness were observed after MCT supplementation in a low-fat diet (medium-chain fatty acids [MCFAs] not specified; 30 g/day of MCTs plus 15 g/day of natural fat) for several years (42).

The difference in the effects of MCT supplementation between patients with TGCV and NLSDM might be explained as follows. (1) A high dose of MCTs was ineffective in reducing fat deposits in cells because a relatively large amount of MCT supplementation over the long term might lead to lipid accumulation in myocytes. (2) MCTs might be more effective in patients suffering effects on the heart than skeletal muscles because the heart might utilize more energy from fatty acids than the skeletal muscles do. (3) The effects of MCTs might depend on the patient’s lifestyle or environment. Therefore, there were large variations in the effects of MCTs between patients, possibly due to confounding factors. These possible effects of MCTs on muscles in patients with TGCV and NLSDM may be applied to treating sarcopenia.

### 4.6 Limitations

This study has several limitations. We estimated muscle size by an anthropometric analysis; however, other modalities such as computed tomography, magnetic resonance imaging, and dual-energy X-ray absorptiometry may be required to confirm the results. The increase in muscle mass might reflect the accumulation of intramuscular lipids rather than an increase in muscle cell number or size; therefore, a muscle biopsy may be necessary. Critical confounding factors might have been missed. For example, ANCOVA might include individual physical activity levels as a covariate. Although a combined data analysis increased the number of participants, even more participants might be needed to observe the significant effects of MCTs in some measures relative to LCTs. Because this study targeted only frail older Japanese individuals, we should have addressed whether similar favorable effects of MCTs would be observed in Western populations with larger body sizes or non-frail subjects. Knowledge of the adverse effects of MCTs is also necessary.

## 5 Conclusion

A combined data analysis of clinical studies concluded that relative to LCTs, chronic supplementation with a low dose (6 g/day) of MCTs (C8:0 and C10:0) in frail older adults increased muscle mass and function. In contrast, it decreased fat mass while maintaining or increasing body weight. These findings indicate the potential for the practical use of MCTs in daily life in treating sarcopenia in older adults. Clinical trials in other groups of frail older adults will be required to verify these favorable effects of MCTs.

## Data availability statement

The data analyzed in this study is subject to the following licenses/restrictions: The data presented in this article may be available on request from the corresponding author in accordance with appropriate data transfer and use agreements. Requests to access these datasets should be directed to ezaki1952@yahoo.co.jp.

## Ethics statement

The studies involving humans were approved by Dr. Teruhisa Yamamoto; The Human Ethics Committee of Showa Women’s University. The studies were conducted in accordance with the local legislation and institutional requirements. Written informed consent for participation in this study was provided by the participants’ legal guardians/next of kin.

## Author contributions

OE: Conceptualization, Formal analysis, Investigation, Writing – original draft. SA: Data curation, Methodology, Writing – review & editing.

## Glossary

AC: mid-upper-arm circumference
ADL: activities of daily living
AMA: mid-upper-arm muscle area
ANCOVA: analysis of covariance
ATGL: adipose triglyceride lipase
BMI: body mass index
CC: calf circumference
GH: growth hormone
KO: knockout
LCFAs: long-chain fatty acids
LCTs: long-chain triglycerides
LCT group: participants receiving 6 g LCTs/day only
LD + LCT: leucine and cholecalciferol-enriched supplement with 6 g LCTs/day
LD + LCT group: participants receiving a leucine and cholecalciferol-enriched supplement with 6 g LCTs/day
LD + MCT: leucine and cholecalciferol-enriched supplement with 6 g MCTs/day
LD + MCT group: participants receiving a leucine and cholecalciferol-enriched supplement with 6 g MCTs/day
MCFAs: medium-chain fatty acids
MCTs: medium-chain triglycerides
MCT group: participants receiving 6 g MCTs/day only
NLSDM: neutral lipid storage disease with myopathy
TGCV: triglyceride deposit cardiomyovasculopathy
TSF: triceps skinfold thickness
