# Supplementary material for: Medium-chain triglycerides (8:0 and 10:0) increase muscle mass and function in frail older adults: a combined data analysis of clinical trials
Source: Front Nutr. 2023 Dec 4;10:1284497. doi: 10.3389/fnut.2023.1284497 (PMC10725933; doi:10.3389/fnut.2023.1284497)
Supplement: Supplementary file 1 [file Table_1.pdf]

Supplementary Table S1. Effect of allocation to trial in combined data analysis; anthropometric measures at baseline and after the 3-mo intervention and their changes from baseline in the MCTs-containing (MCT or LD + MCT) and LCTs-containing (LCT or LD + LCT) groups (*n* = 56)[1].

**Measure**	**Group**	***n***	**Baseline**	**3-mo intervention**	**Non-adjusted change**	**Adjusted change for baseline[3]**	**Adjusted change for trial[4]**	**Adjusted change for baseline & trial[5]**
Body weight, kg	MCT, LD + MCT	29	43.4 ± 10.6	44.6 ± 10.1***	1.2 ± 1.7	1.2 (0.6, 1.8)	1.2 (0.6, 1.8)	1.2 (0.6, 1.8)
	LCT, LD + LCT	27	43.6 ± 5.8	43.8 ± 5.4	0.2 ± 1.6	0.2 (-0.4, 0.8)	0.2 (-0.4, 0.9)	0.2 (-0.4, 0.8)
P value[2]					0.026	0.021	0.027	0.022
BMI, kg/m^2	MCT, LD + MCT	29	18.5 ± 3.4	19.1 ± 3.4***	0.6 ± 0.8	0.5 (0.3, 0.8)	0.6 (0.3, 0.8)	0.5 (0.3, 0.8)
	LCT, LD + LCT	27	19.2 ± 1.9	19.3 ± 1.7	0.1 ± 0.7	0.1 (-0.2, 0.4)	0.1 (-0.2, 0.4)	0.1 (-0.2, 0.4)
P value					0.027	0.042	0.028	0.044
Right AC, cm	MCT, LD + MCT	29	22.0 ± 3.6	22.4 ± 3.7**	0.5 ± 0.9	0.5 (0.1, 0.8)	0.5 (0.1, 0.8)	0.4 (0.1, 0.8)
	LCT, LD + LCT	26	22.8 ± 2.1	22.8 ± 1.8	-0.0 ± 0.9	-0.0 (-0.4, 0.3)	-0.0 (-0.4, 0.3)	-0.0 (-0.4, 0.3)
P value					0.039	0.06	0.035	0.05
Left AC, cm	MCT, LD + MCT	29	21.9 ± 3.5	22.3 ± 3.5	0.4 ± 0.6	0.4 (0.2, 0.7)	0.4 (0.2, 0.7)	0.4 (0.2, 0.7)
	LCT, LD + LCT	26	22.4 ± 2.5	22.7 ± 2.2	0.3 ± 0.9	0.3 (-0.0, 0.6)	0.2 (-0.0, 0.5)	0.3 (-0.0, 0.6)
P value					0.35	0.44	0.35	0.44
Right TSF, mm	MCT, LD + MCT	29	10.2 ± 5.2	8.8 ± 4.7*	-1.4 ± 3.1	-1.4 (-2.4, -0.4)	-1.4 (-2.5, -0.3)	-1.4 (-2.4, -0.4)
	LCT, LD + LCT	26	10.4 ± 4.9	11.3 ± 4.7	0.9 ± 2.9	0.9 (-0.1, 2.0)	0.9 (-0.3, 2.1)	1.0 (-0.1, 2.0)
P value					0.007	0.003	0.007	0.003
Left TSF, mm	MCT, LD + MCT	29	8.3 ± 5.3	7.6 ± 4.7	-0.7 ± 2.7	-0.7 (-1.7, 0.4)	-0.7 (-1.8, -0.4)	-0.7 (-1.7, 0.4)
	LCT, LD + LCT	26	8.4 ± 4.3	8.8 ± 4.8	0.4 ± 3.2	0.4 (-0.7, 1.5)	0.4 (-0.7, 1.6)	0.4 (-0.7, 1.6)
P value					0.18	0.16	0.18	0.15
Calculated right	MCT, LD + MCT	29	28.6 ± 6.0	30.3 ± 5.7**	1.7 ± 2.5	1.4 (0.5, 2.3)	1.7 (0.7, 2.7)	1.4 (0.6, 2.3)
AMA, cm^2	LCT, LD + LCT	26	30.7 ± 5.3	29.8 ± 4.0	-0.9 ± 2.7	-0.7 (-1.6, 0.3)	-0.9 (-2.0, 0.1)	-0.7 (-1.5, 0.2)
P value					<0.001	0.002	<0.001	0.001

Calculated left AMA, cm^2	MCT, LD + MCT	29	29.1 ± 5.1	30.8 ± 5.4	1.7 ± 3.4	1.6 (0.5, 2.7)	1.7 (0.6, 2.8)	1.6 (0.5, 2.7)
	LCT, LD + LCT	26	30.8 ± 4.7	31.1 ± 5.1	0.4 ± 2.5	0.5 (-0.7, 1.6)	0.4 (-0.8, 1.5)	0.5 (-0.7, 1.7)
P value					0.10	0.16	0.10	0.16
Right CC, cm	MCT, LD + MCT	28	28.8 ± 4.3	29.0 ± 4.4	0.2 ± 1.0	0.2 (-0.2, 0.6)	0.2 (-0.2, 0.7)	0.2 (-0.2, 0.6)
	LCT, LD + LCT	25	28.9 ± 3.1	28.9 ± 2.8	-0.1 ± 1.2	-0.0 (-0.5, 0.4)	-0.1 (-0.5, 0.4)	-0.0 (-0.5, 0.4)
P value					0.34	0.36	0.36	0.36
Left CC, cm	MCT, LD + MCT	28	28.5 ± 4.5	28.8 ± 4.5	0.3 ± 0.8	0.3 (-0.2, 0.7)	0.3 (-0.2, 0.7)	0.3 (-0.2, 0.7)
	LCT, LD + LCT	25	28.5 ± 2.8	28.3 ± 2.4	-0.2 ± 1.5	-0.2 (-0.6, 0.3)	-0.2 (-0.7, 0.3)	-0.2 (-0.7, 0.3)
P value					0.16	0.16	0.17	0.16

[1]Values are means ± SD or adjusted mean (95% CI). Difference from baseline by Wilcoxon signed-rank test, $^{*}P < 0.05$, $^{**}P < 0.01$, $^{***}P < 0.001$.
[2]*P* value represents the differences in the changes of variables between the 2 groups assessed by 1-factor ANCOVA, non-adjusted, [3]adjusted for each baseline value (model 1), [4]adjusted for allocation to trial (Trial 1 or 2), and [5]adjusted for baseline values of each measurement and allocation to trial.
AC, arm circumference; AMA, arm muscle area; BMI, body mass index; CC, calf circumference; LCT, 6 g/d of long-chain triglycerides; LD + LCT, leucine and cholecalciferol-enriched supplement with 6 g/d of long-chain triglycerides; LD + MCT, leucine and cholecalciferol-enriched supplement with 6 g/d of medium-chain triglycerides; MCT, 6 g/d of medium-chain triglycerides; TSF, triceps skinfold thickness.
