# Supplementary material for: Medium-chain triglycerides (8:0 and 10:0) increase muscle mass and function in frail older adults: a combined data analysis of clinical trials
Source: Front Nutr. 2023 Dec 4;10:1284497. doi: 10.3389/fnut.2023.1284497 (PMC10725933; doi:10.3389/fnut.2023.1284497)
Supplement: Supplementary file 2 [file Table_2.pdf]

Supplementary Table S2. Effect of allocation to trial in combined data analysis; muscle strength and function at baseline and after the 3-mo intervention and their changes from baseline in the MCTs-containing (MCT or LD + MCT) and LCTs-containing (LCT or LD + LCT) groups (n = 56)[1].

Measure	Group	*n*	Baseline	3-mo intervention	Non-adjusted change	Adjusted change for baseline[3]	Adjusted change for trial[4]	Adjusted change for baseline & trial[5]
Right-hand grip strength, kg	MCT, LD + MCT	29	11.3 ± 7.6	13.0 ± 7.2***	1.7 ± 2.0	1.7 (1.1, 2.3)	1.7 (1.1, 2.3)	1.7 (1.1, 2.3)
	LCT, LD + LCT	25	11.7 ± 5.1	11.8 ± 4.9	0.2 ± 1.3	0.2 (-0.5, 0.8)	0.1 (-0.5, 0.8)	0.2 (-0.5, 0.8)
P value[2]					0.002	0.001	0.001	<0.001
Left-hand grip strength, kg	MCT, LD + MCT	27	10.2 ± 4.6	11.4 ± 4.5	1.3 ± 2.6	1.2 (0.3, 2.1)	1.3 (0.3, 2.2)	1.2 (0.3, 2.2)
	LCT, LD + LCT	25	10.8 ± 5.4	11.6 ± 5.5	0.8 ± 2.3	0.8 (-0.1, 1.8)	0.8 (-0.2, 1.8)	0.8 (-0.1, 1.8)
P value					0.51	0.57	0.51	0.57
Walking speed, m/s	MCT, LD + MCT	23	0.637 ± 0.409	0.706 ± 0.427**	0.069 ± 0.124	0.068 (0.022, 0.113)	0.069 (0.025, 0.113)	0.068 (0.022, 0.113)
	LCT, LD + LCT	20	0.436 ± 0.257	0.420 ± 0.285	-0.016 ± 0.073	-0.014 (-0.062, 0.035)	-0.016 (-0.063, 0.032)	-0.014 (-0.063, 0.035)
P value					0.011	0.020	0.012	0.021
Legs open and close test, n/10 s	MCT, LD + MCT	28	4.14 ± 3.13	6.48 ± 3.90***	2.34 ± 1.88	2.32 (1.67, 2.97)	2.34 (1.70, 2.99)	2.32 (1.67, 2.98)
	LCT, LD + LCT	22	5.80 ± 2.89	4.96 ± 2.59*	-0.84 ± 1.35	-0.82 (-1.56, -0.08)	-0.85 (-1.57, -0.12)	-0.82 (-1.57, -0.08)
P value					< 0.001	<0.001	<0.001	<0.001
Peak expiratory flow, L/min	MCT, LD + MCT	25	189 ± 83	220 ± 82**	31 ± 48	32 (13, 52)	32 (13, 51)	33 (15, 52)
	LCT, LD + LCT	26	173 ± 66	179 ± 75	6 ± 50	5 (-14, 24)	5 (-13, 24)	4 (-14, 22)
P value					0.08	0.049	0.045	0.028

[1]Values are means ± SD or adjusted mean (95% CI). Difference from baseline by Wilcoxon signed-rank test, $^{*}P < 0.05$, $^{**}P < 0.01$, $^{***}P < 0.001$.

[2]*P* value represents the differences in the changes of variables between the 2 groups assessed by 1-factor ANCOVA, [3]adjusted for each baseline value (model 1), [4]adjusted for allocation to trial (Trials 1 or 2), and [5]adjusted for baseline values of each measurement and allocation to trial.

LCT, 6 g/d of long-chain triglycerides; LD + LCT, leucine and cholecalciferol-enriched supplement with 6 g/d of long-chain triglycerides; LD + MCT, leucine and cholecalciferol-enriched supplement with 6 g/d of medium-chain triglycerides; MCT, 6 g/d of medium-chain triglycerides.
